# Supplementary material for: Metabolomic Profiling Identifies Key Metabolites and Defense Pathways in Rlm1-Mediated Blackleg Resistance in Canola
Source: Int J Mol Sci. 2025 Jun 12;26(12):5627. doi: 10.3390/ijms26125627 (PMC12192793; doi:10.3390/ijms26125627)
Supplement: Supplementary file 1 [file ijms-26-05627-s001.zip › ijms-3595597-supplementary/Supple Figures.pdf]

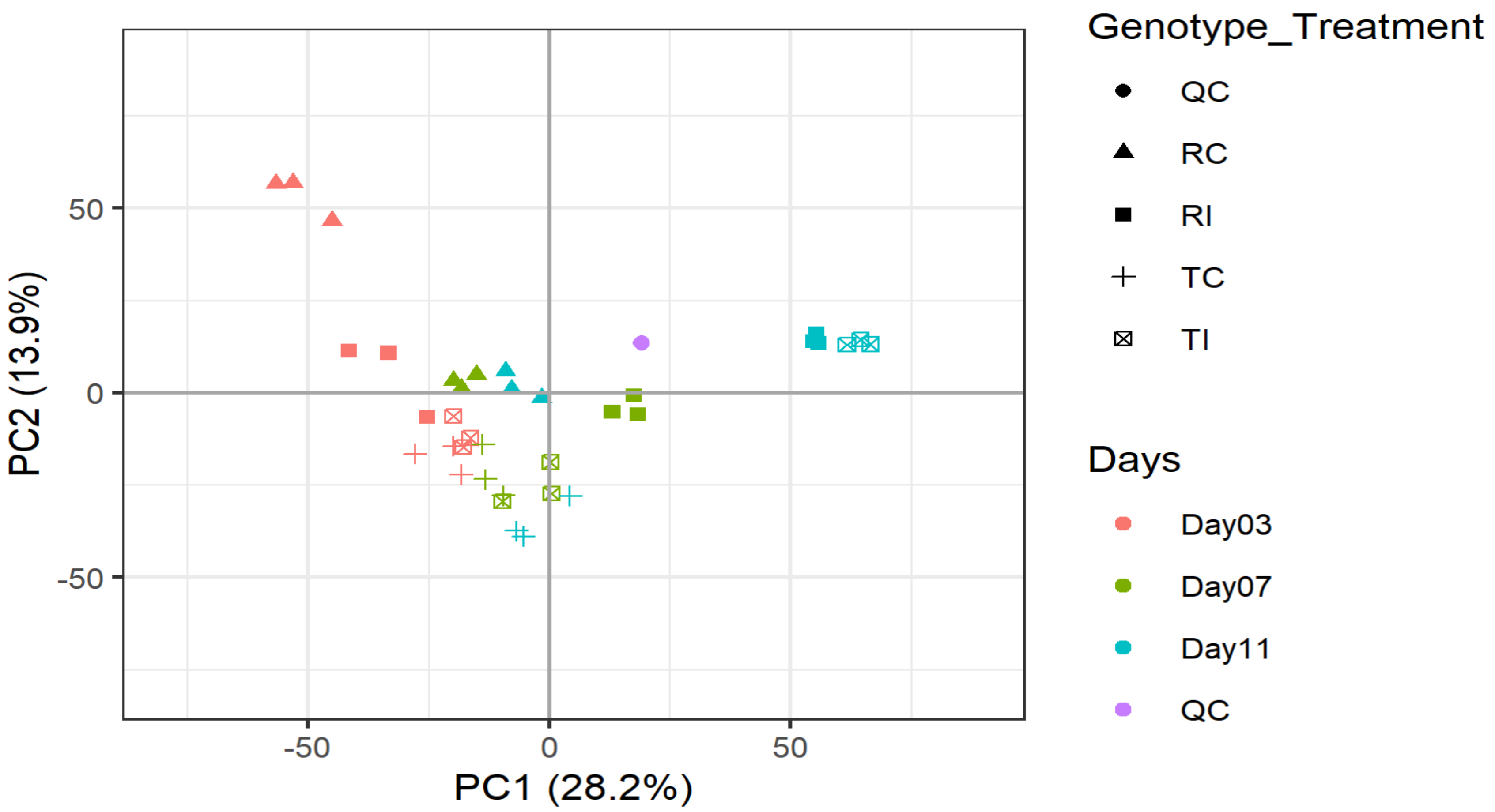

Figure S1 PCA analysis of metabolomic data over Topas (T) and Topas-Rlm1 (R) lines prick inoculated with water (control -C) or *L. maculans* (*AvrLm1* -I) sampled at 3, 7 and 11 days after inoculation (dai). QC denotes quality-control samples.

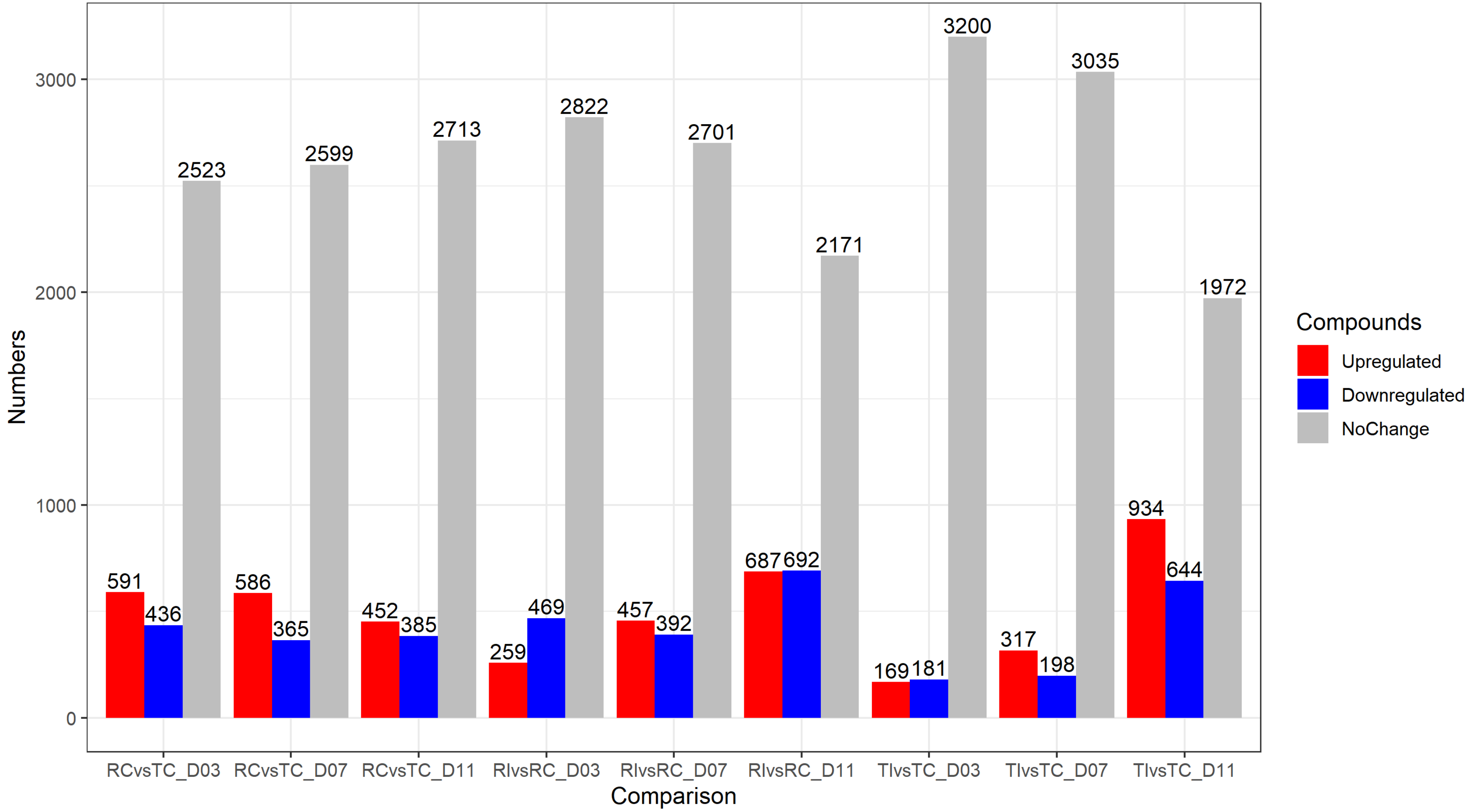

Figure S2 The number of significantly up-accumulated (red bars) and down-accumulated (blue bars) metabolites identified in Topas (T) and Topas-Rlm1 (R) with water (C) or *L. maculans* (I) inoculation sampled at 3, 7 and 11 days after inoculation (dai). For example, RCvsTC_D03 on the horizontal axis compares Topas-Rlm1 control with Topas control

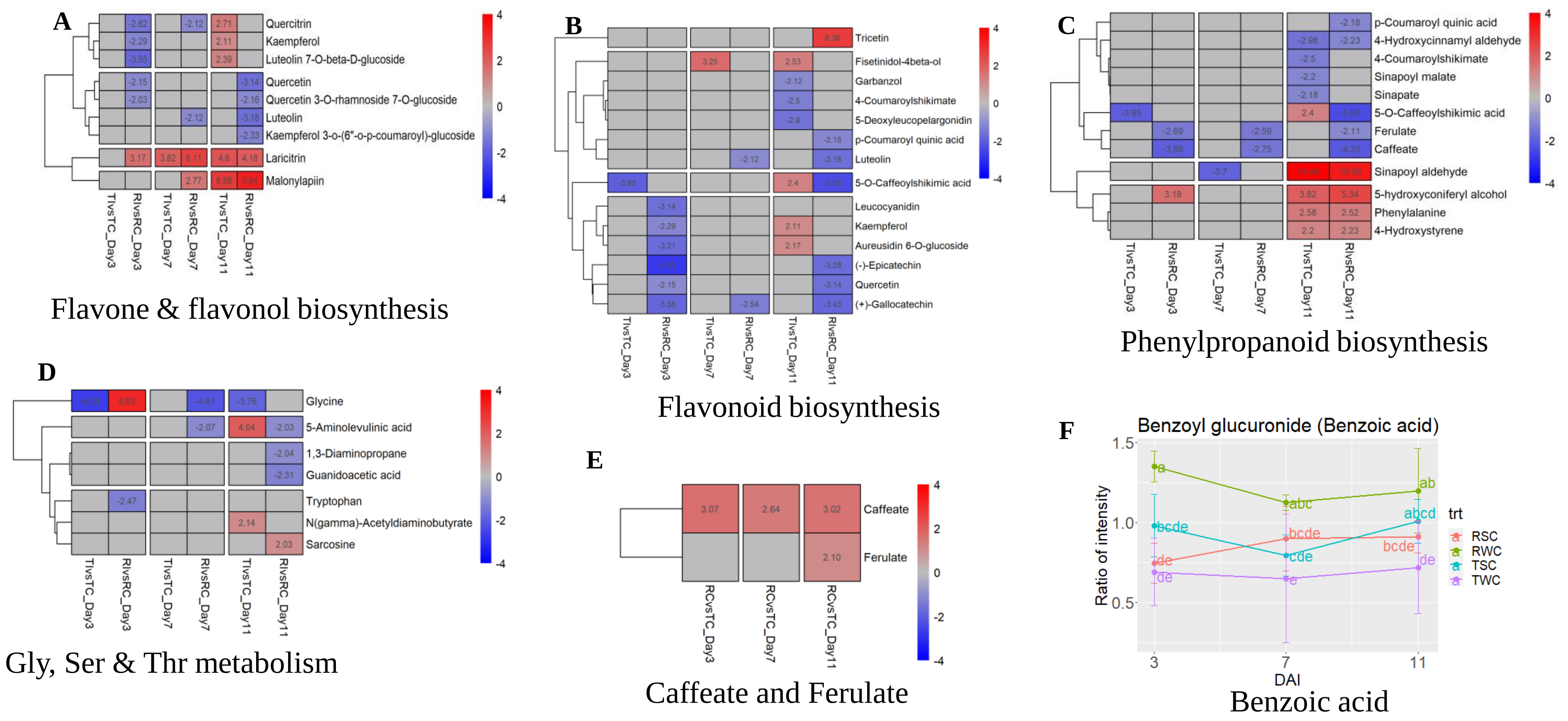

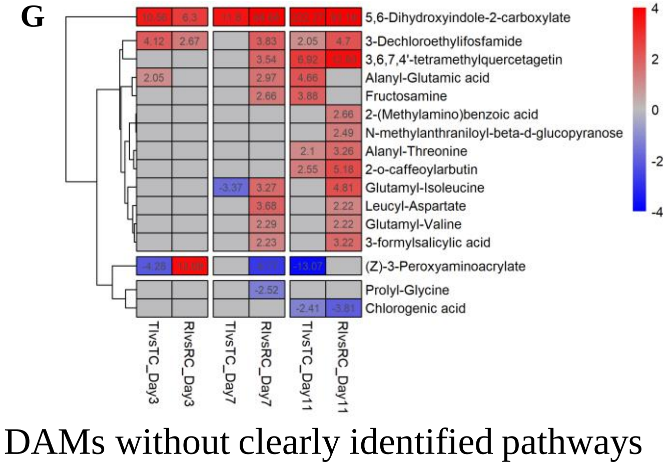

Figure S3 A-F) Accumulation of metabolites associated with additional amino acid pathways and plant responses to abiotic stresses. G) Several highly-regulated DAMs were not associated with any known pathways.

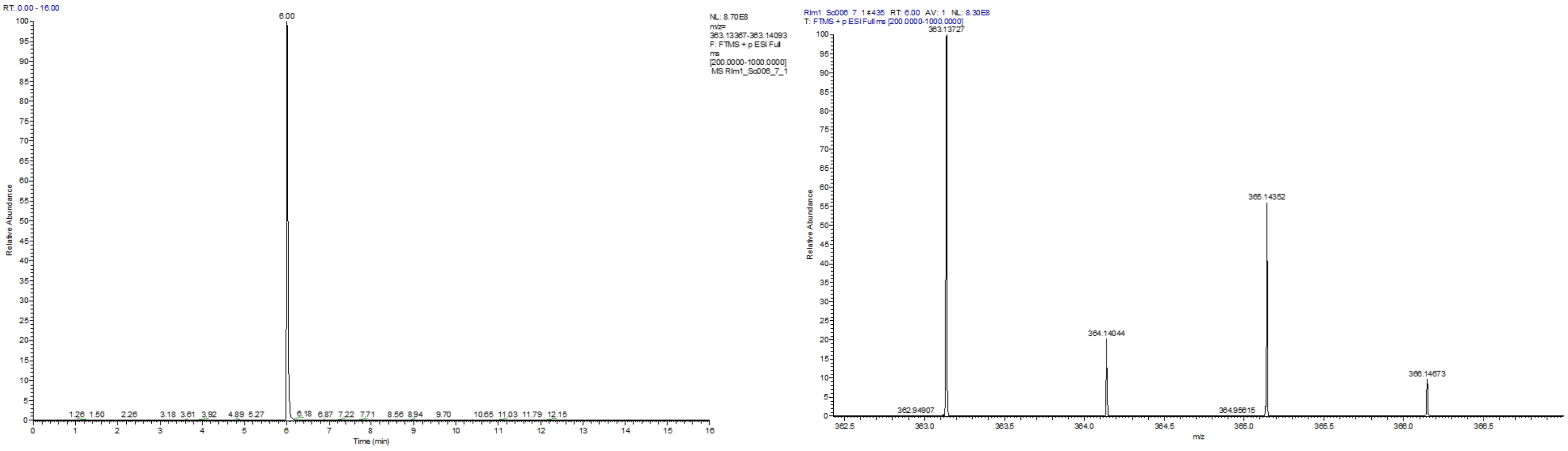

Figure S4. Extracted Ion Chromatogram and Mass Spectrum of Pipecolic Acid

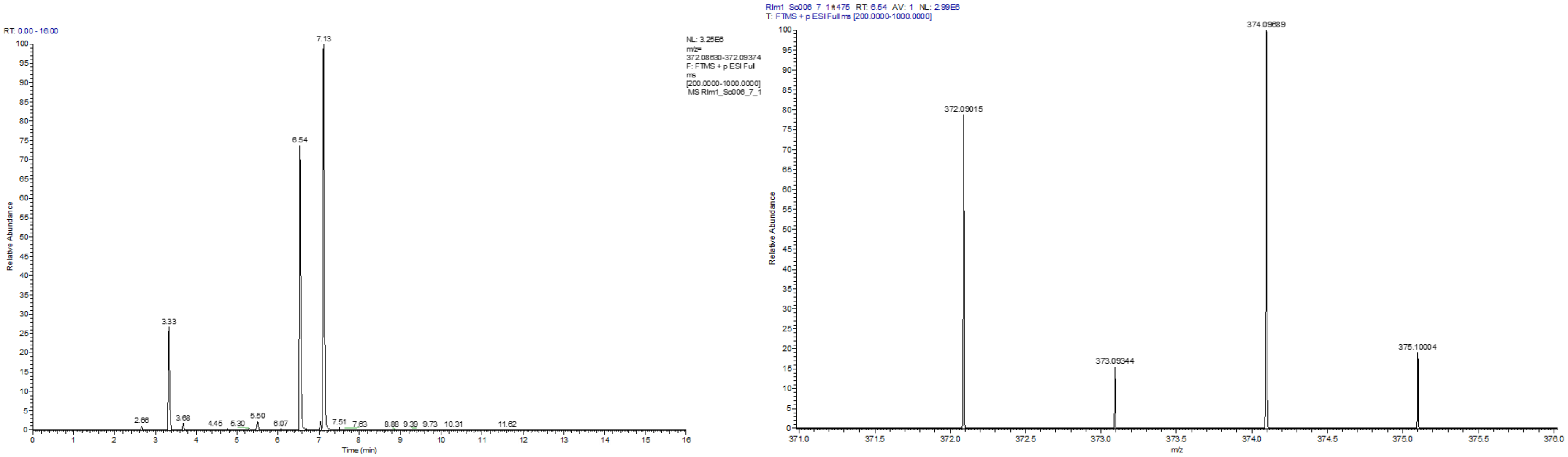

Figure S5. Extracted Ion Chromatogram and Mass Spectrum of Salicylic Acid

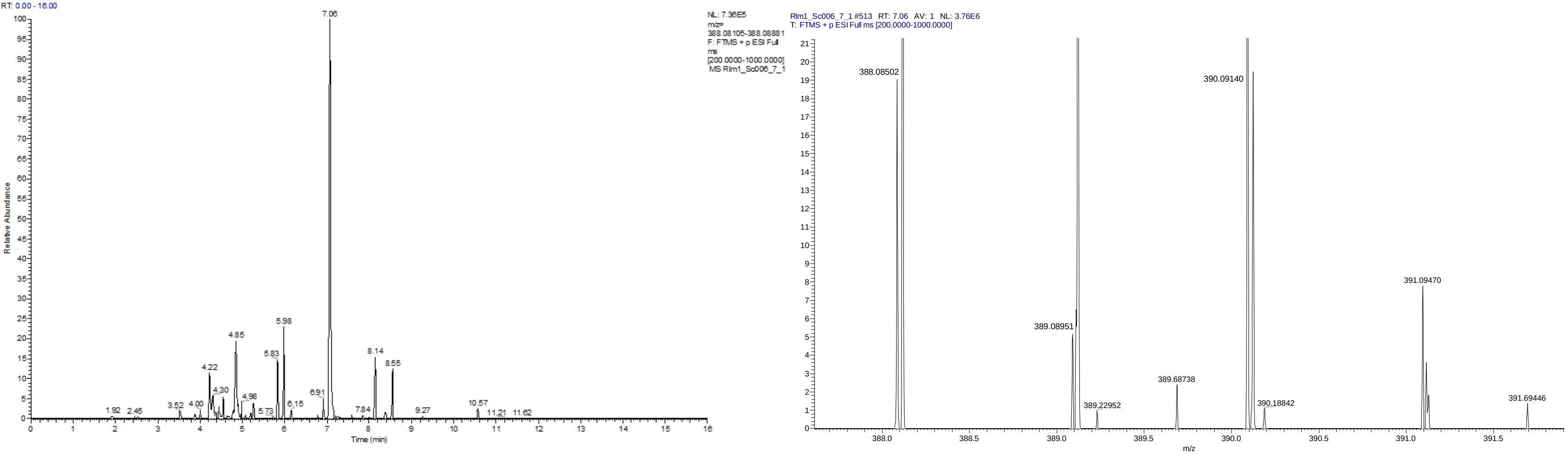

Figure S6. Extracted Ion Chromatogram and Mass Spectrum of Gentisic Acid

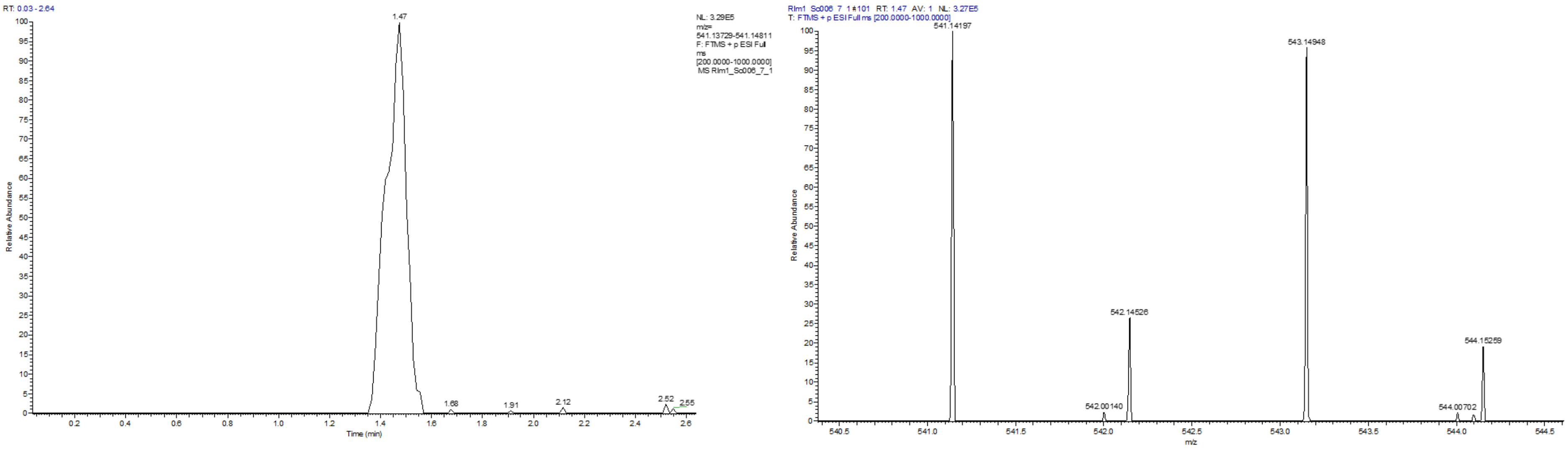

Figure S7. Extracted Ion Chromatogram and Mass Spectrum of Glutathione

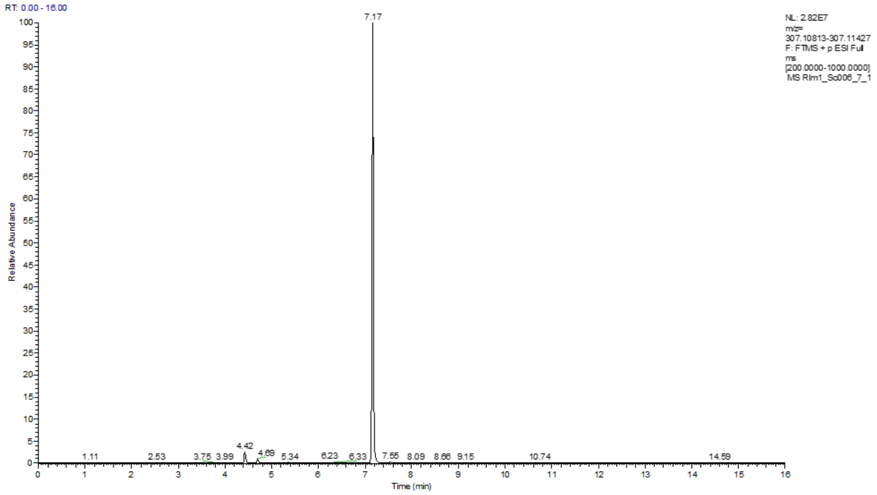

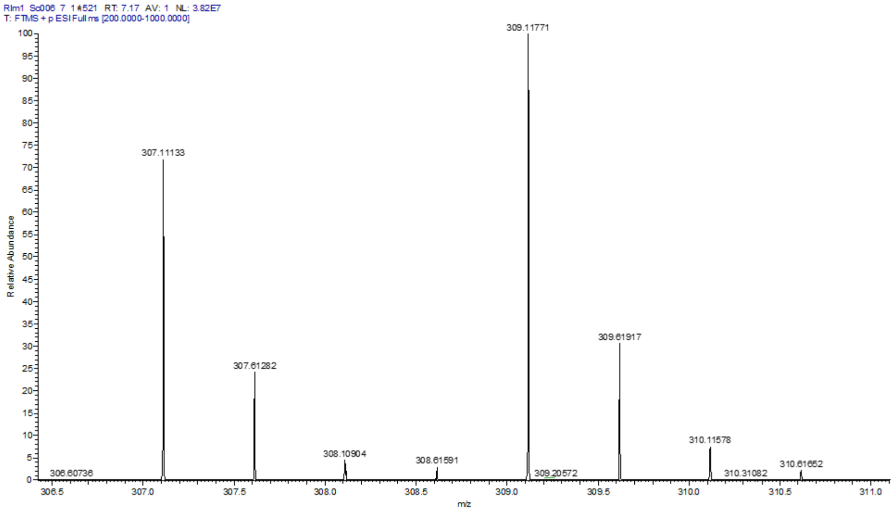

Figure S8. Extracted Ion Chromatogram and Mass Spectrum of Lysine

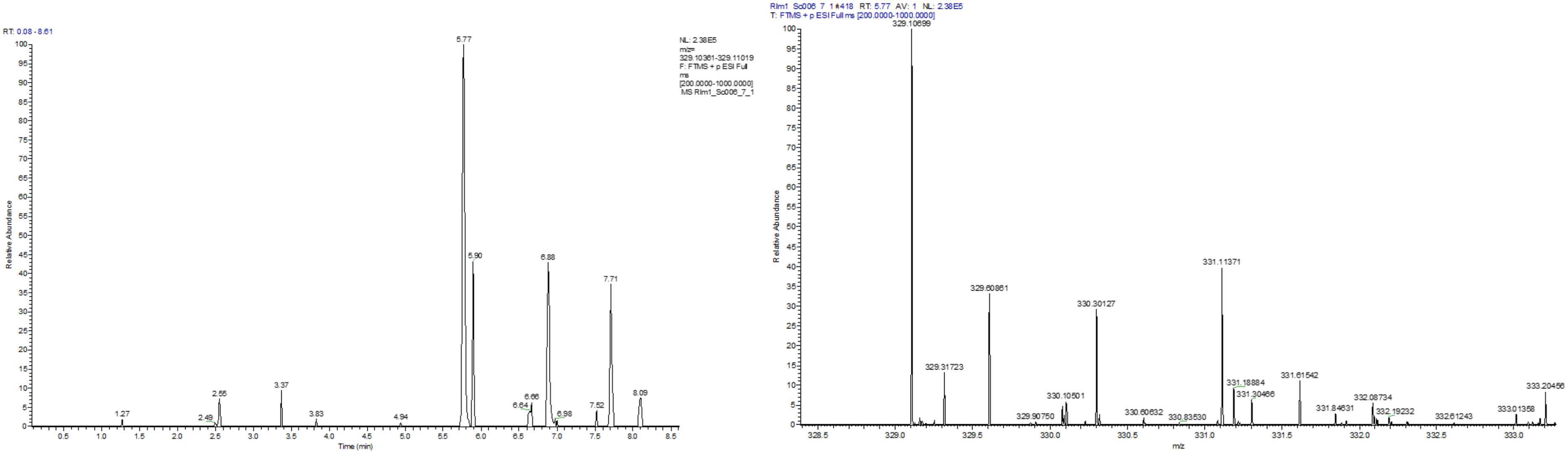

Figure S9. Extracted Ion Chromatogram and Mass Spectrum of Diaminopimelic Acid

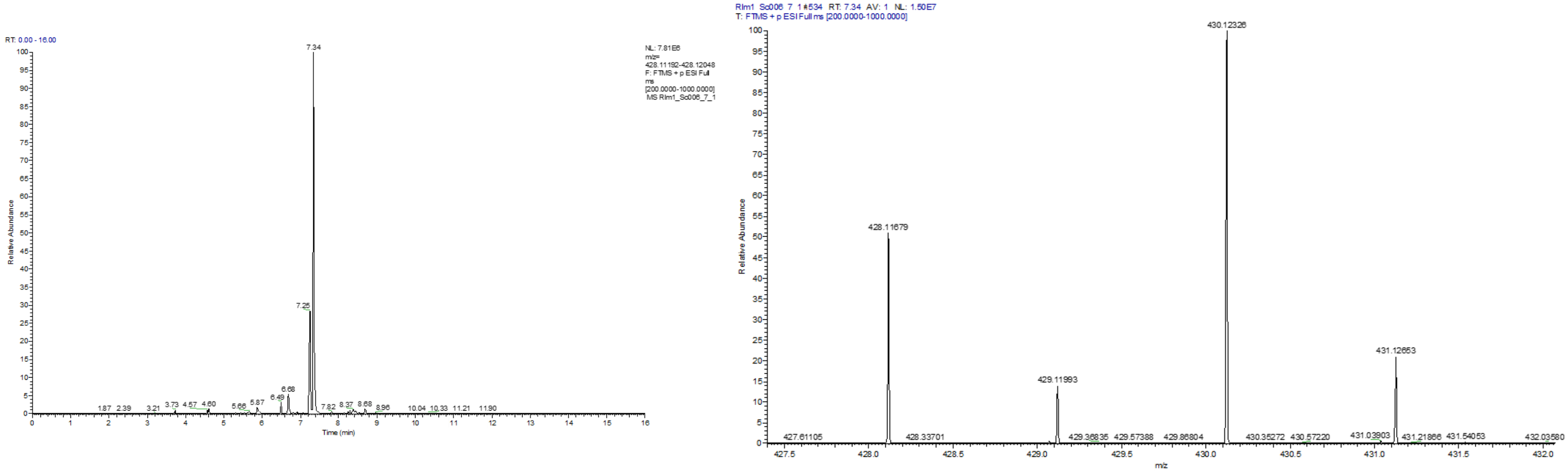

Figure S10. Extracted Ion Chromatogram and Mass Spectrum of Ferulic Acid

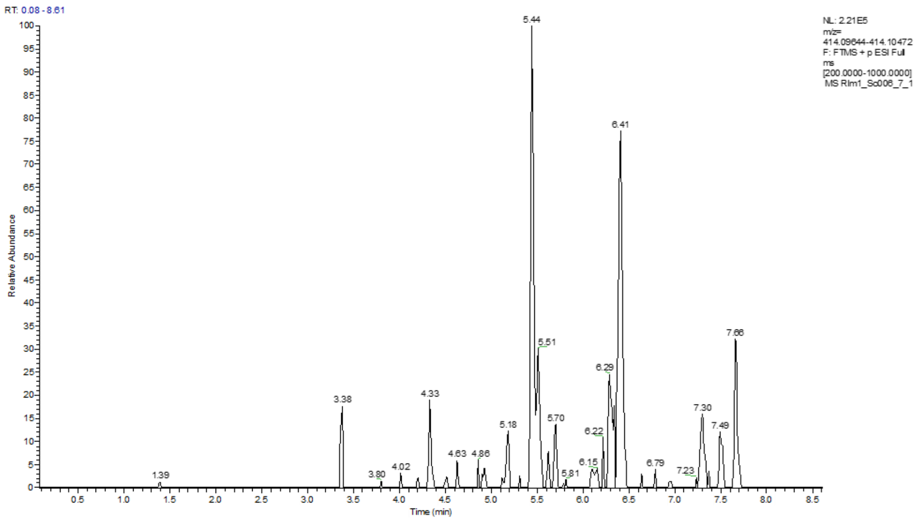

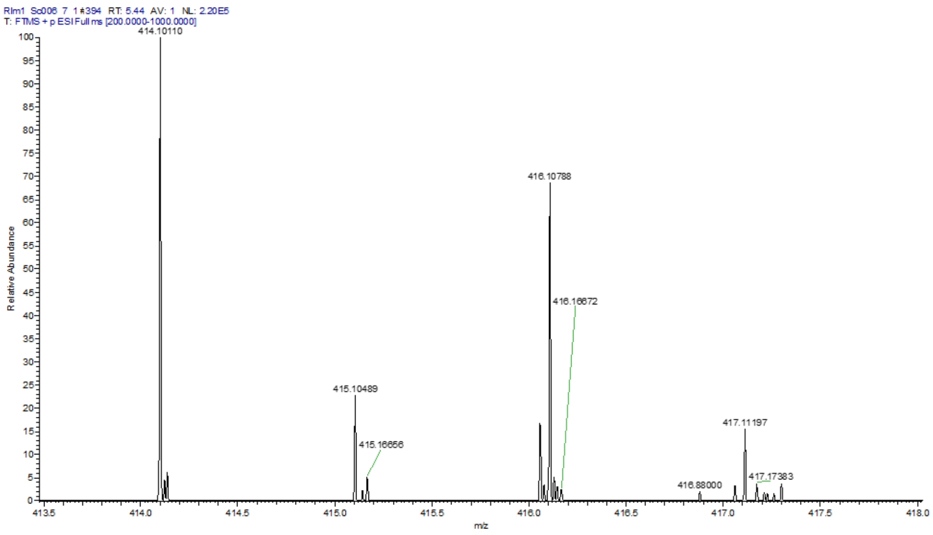

Figure S11. Extracted Ion Chromatogram and Mass Spectrum of Caffeic Acid
